# Elements of Materia Medica and Therapeutics

**Published:** 1846-08

**Authors:** 


ARTICLE VII.

*Elements of Materia Medica and Therapeutics.* By Edward Ballard, M. D., London, Physician to St. Pancras Royal General Dispensary, and Medical Tutor in University College, London, and Alfred Baring Garrod, M.D., London, Physician to the Fore Street Dispensary, and Lecturer on Materia Medica and Therapeutics in the Aldersgate School of Medicine: with additions and alterations by R. Eglesfield Griffith, M. D. Philadelphia: Hogan & Thompson. 1846. 8 vo. pp. 516.

In this work the authors have divested their text of every thing but that which is absolutely essential.

The classification is a natural historical one, the theories of the chemical process are illustrated by diagrams.

The editor has added the more valuable indigenous plants used in Medicine, and has adapted the whole to the United States Pharmacopœia. The arrangement which has been adopted in the description of the individual articles is systematically adhered to throughout, and the whole is written in a clear and concise manner. With the additions and emendations of the editor, it is valuable, especially as a text book for American Students. H. S. H.

